# Fedflow: cloud orchestration for federated learning with the FeatureCloud platform

**DOI:** 10.1093/bioadv/vbag255

**Published:** 2026-09-04

**Authors:** Lukas Weilguny, Niklas Probul, Yani Ren, Nicolas Pons, Jan Baumbach, Mathieu Almeida

**Affiliations:** Université Paris-Saclay, INRAE, MGP, Jouy-en-Josas, F-78350, France; Université Paris-Saclay, INRAE, Micalis Institute, Jouy-en-Josas, F-78350, France; Institute for Computational Systems Biology, Universität Hamburg, Hamburg, Germany; Université Paris-Saclay, INRAE, MGP, Jouy-en-Josas, F-78350, France; Université Paris-Saclay, INRAE, MGP, Jouy-en-Josas, F-78350, France; Institute for Computational Systems Biology, Universität Hamburg, Hamburg, Germany; Department of Mathematics and Computer Science, University of Southern Denmark, Odense, Denmark; Université Paris-Saclay, INRAE, MGP, Jouy-en-Josas, F-78350, France

## Abstract

**Motivation:**

Federated learning (FL) enables collaborative model training on geographically distributed genomic and clinical datasets while complying with data privacy laws and regulatory constraints. FeatureCloud is an existing platform for FL that provides an accessible web-based interface and a large repository of implemented methods. However, due to its graphical interface, FeatureCloud requires manual interaction of all participants, limiting automation, iteration, and reproducibility.

**Results:**

We introduce fedflow, a Python-based command-line tool for headless orchestration of FL tasks with FeatureCloud. This tool uses distributed computing resources such as virtual machines or cloud instances to automate such workflows. This allows for scalable federated computing either in local simulations or deployed in a trusted environment. Further, we demonstrate how fedflow can be used to integrate FeatureCloud in reproducible Snakemake workflows. For this, we reanalyse a metagenomic dataset with two federated algorithms and compare the results to the centralized approach with pooled data. Overall, fedflow enables automation of multi-client FL tasks, facilitates embedding of FeatureCloud in standard bioinformatics pipelines and thereby helps increase reproducibility.

**Availability:**

Fedflow is open-source and available at https://github.com/W-L/fedflow.

## 1 Introduction

Federated learning (FL) is a powerful paradigm to run collaborative machine learning tasks across distributed data without centralizing sensitive information. This addresses a challenge in genomic research: preserving privacy and adhering to regulatory constraints while leveraging genomic datasets and clinical records from diverse sources. On the one hand, large-scale datasets from multiple cohorts enable novel approaches to predictive modeling (e.g. Lee *et al.* 2024, Piccinno *et al.* 2025); on the other hand, privacy laws require that institutional policies and research procedures provide appropriate protection which thus might preclude data pooling (Shabani and Marelli 2019, Wan *et al.* 2022). Federated approaches train models locally at custodial sites by sharing only model parameters or updates and therefore mitigate information leakage risks and respect data sovereignty (Yurdem *et al.* 2024).

Recent studies demonstrate the applicability of FL for genomic prediction tasks across multiple clients with performance close to centralized models on phenotype predictions (e.g. Kolobkov *et al.* 2024). Deploying FL workflows at scale in different settings, however, comes with technical challenges. To address these, a range of software libraries and analysis platforms that implement privacy-preserving algorithms exist, e.g. reviewed in (Riedel *et al.* 2024, Chavero-Diez *et al.* 2026).

To address these, a range of software libraries and analysis platforms for federated learning exists, ranging from general-purpose frameworks to domain-specific solutions (Riedel *et al.* 2024, Chavero-Diez *et al.* 2026). Widely-used systems include Flower (Beutel *et al.* 2022), NVIDIA FLARE (Roth *et al.* 2022), and PySyft (Ziller *et al.* 2021). They emphasize flexibility and extensibility by wrapping and extending various machine-learning libraries, allowing users to define custom training and aggregation strategies, and are intended to build bespoke federated applications.

FeatureCloud, which we introduced recently (Matschinske *et al.* 2023), occupies a distinct niche in this landscape. Rather than serving as a general-purpose development kit for constructing federated protocols, it focuses specifically on accessibility of federated biomedical analyses, with a unified interface for ready-to-use federated applications (Matschinske *et al.* 2023). This app-centric design is intended to lower the barrier to applying federated methods in biomedical studies by abstracting some of the complexities of distributed computation and secure communication. However, the web-based platform introduces limitations: all participants are required to manually interact with the graphical interface, prohibiting automation and iteration of analyses. Additionally, embedding FL tasks with FeatureCloud in reproducible workflows is currently not possible.

We therefore introduce fedflow, a complementary tool to automate FL tasks with FeatureCloud. Our tool orchestrates remote machines to allow for reproducible analyses while relying on an established framework. In other words, fedflow contributes scalability and reproducibility of multi-client executions, while the underlying statistical and information flow models remain those of the selected FeatureCloud apps. For this, fedflow consists of (i) the orchestration of distributed computing resources (e.g. virtual machines, cloud instances, or bare-metal) to automate FL, and (ii) an API to interact headlessly with FeatureCloud. Finally, we present an example of how this tool can be used to embed FeatureCloud within reproducible Snakemake workflows, addressing its current limitations and enhancing reproducibility.

## 2 Implementation

The main motivation for fedflow is to enable integration of FeatureCloud in programmatic workflows and eliminate manual interaction with its web-based interface. Accordingly, fedflow should be understood as workflow-oriented automation framework for FeatureCloud, not as a general-purpose federated learning library. Fedflow is designed as a CLI tool and implemented in python; installed via pip install fedflow-featurecloud.

### 2.1 Orchestration of remote machines

**Figure 1:**
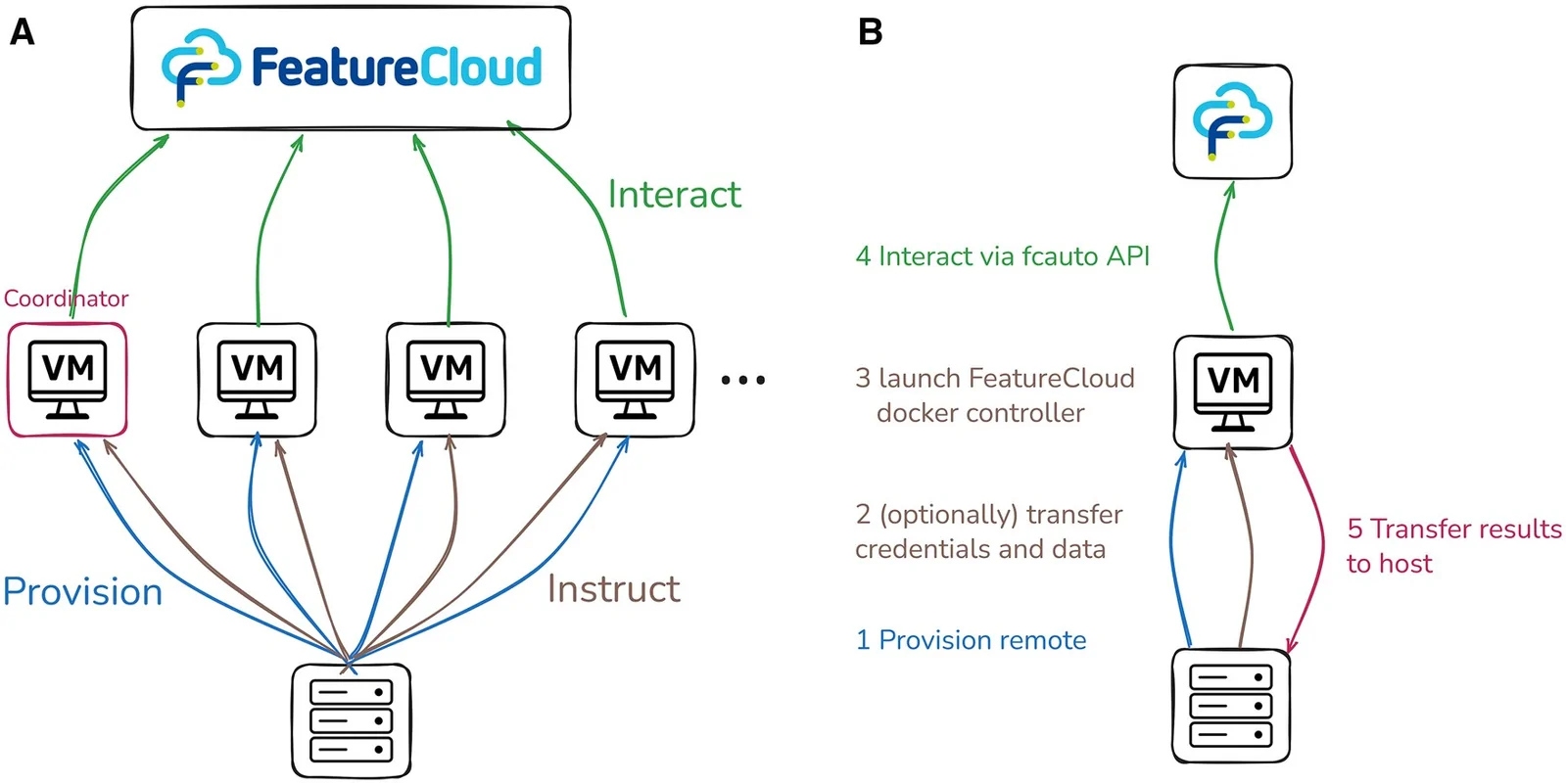
Overview of fedflow. (A) The orchestrator is executed on a host machine, which connects to clients via ssh. The clients are provisioned automatically and instructed to interact with the FeatureCloud platform to run federated learning tasks. (B) On each client acting as a participant in a FeatureCloud project, system dependencies are installed, credentials and data are optionally transferred from the host machine, and the local FeatureCloud docker controller is started. Interactions with FeatureCloud itself are automated with the fcauto API. Finally, the output of each client can be transferred to the host machine to gather analysis results. Exchange of credentials, data, and results is implemented for convenience during simulations and testing.

A typical FeatureCloud analysis consists of multiple participants—research institutes, hospitals, or other data holders—contributing their local information to some shared FL task. Here, we replace individually-acting participants by clients that are orchestrated via a host machine (Fig. 1). These clients can be locally-managed VMs, cloud instances, participants’ bare-metal machines, or other distributed computing resources accessible via ssh.

Fedflow is configured using a single toml file, which determines the executed FeatureCloud app and contains information about the clients. More specifically, an array of [[clients]] in the toml file is used to declare the participants, i.e. the acting FeatureCloud users, and ssh connection details to the respective compute instances. Ssh connection details can also be omitted, in which case fedflow starts local VMs on the host machine instead; using vagrant and libvirt (HashiCorp 2025, libvirt community 2025). In this case clients remain isolated—albeit on the same hardware—allowing for realistic simulations of federated execution.

The configuration file also contains a list of data contributed to the FeatureCloud task by each client. For federated analyses, participating sites generally need to provide data in a compatible representation using a shared data model and harmonized schema, such that the selected FeatureCloud app can interpret inputs consistently across clients. Fedflow itself does not define or validate a statistical data model. Instead, it assumes that each selected FeatureCloud app receives inputs in the representation expected by that app and that equivalent preprocessing has been applied across sites. This includes consistent variable definitions, encoding of categorical variables and missing values, and any centering, scaling, or other transformation required for the federated method. This is particularly important when cohorts differ in covariate distributions, class balance, or other site-specific characteristics, since such heterogeneity can affect local model fitting and the behavior of the resulting federated model. Fedflow orchestrates data delivery and execution, but it does not infer, harmonize, or validate these statistical assumptions. However, by allowing the integration of FeatureCloud in workflows, fedflow can help ensure that preprocessing is performed consistently across participating sites. The data can either be present on the client, or can be automatically distributed to the participants from the host for simulation purposes alongside user credentials for the FeatureCloud platform. A template configuration file can be generated by running fedflow -t.

All communication of host and clients is implemented with the fabric library (fabric developers 2026). More specifically, instructions are sent one-way to control client behaviour via ssh remote execution, specifically via fabric’s ThreadingGroup for concurrency. The only instance of information flow from clients to the host is the optional retrieval of results as a final step. Fedflow automatically provisions remotes in an idempotent manner, i.e. setting up system dependencies needed for FeatureCloud FL executions, including docker. Client-locally FeatureCloud operates a docker container, termed featurecloud controller, which supervises local training and communicates with the FeatureCloud platform backend. Additionally, the controller instance oversees inter-client exchange via the FeatureCloud relay server (Matschinske *et al.* 2023). Following these setup steps, fedflow instructs the clients to contribute their local data and launch FL tasks via an API. Fedflow is intended for automation in trusted environments and should not be interpreted as a standalone security or privacy-preserving framework.

### 2.2 Headless communication with FeatureCloud

To enable automated FL across remotes, we implemented fcauto, a lightweight Python-based API for headless interaction between clients and the FeatureCloud platform. FeatureCloud users, projects, and apps are encapsulated as objects for authentication on the platform, creating or joining federated projects, and managing project execution states. The API abstracts both the FeatureCloud endpoints and the client-local docker controller, thereby providing a single layer to control FL execution. In other words, fcauto implements both platform-level operations (e.g. project creation, participant management, app configuration, and project state changes), and controller-level operations, including contribution of training input and tracking execution progress. This mirrors FeatureCloud’s architectural separation but means that clients can be orchestrated with a single interface. The fcauto API exposes its functionality as a library and through a command-line interface.

Fedflow uses this API internally to instruct all clients to interact with FeatureCloud and their local docker controller. For example, fedflow would send the CLI call fcauto contribute -u < featurecloud_user> -p < project_id> -d < data_paths> as an instruction over ssh to a client in order for it to authenticate with the platform and contribute its data to the local controller for execution of a specific project. As soon as all contributions have been registered, the client-local controllers start the FL tasks. A client designated as project coordinator monitors the project execution through periodic status queries. Fedflow does not implement federated aggregation algorithms itself; these are defined by the selected FeatureCloud app and can therefore differ across tasks. Upon completion, outputs from federated runs are retrieved directly from the local controller’s directory structure facilitating downstream analysis. fcauto can also be used for headless interaction with FeatureCloud outside of the orchestration tool presented here.

## 3 Federated learning within reproducible workflows

To demonstrate how fedflow enables the integration of FeatureCloud in bioinformatics workflows, we present an end-to-end federated analysis of a publically-available dataset using Snakemake. This workflow combines dimensionality reduction and supervised learning in both centralized and federated settings. To reduce technical heterogeneity across clients, we used a curated dataset with harmonized sample processing and batch-effect corrected metagenomic species abundances as input (Barbet *et al.* 2025). This preprocessing is regarded as a prerequisite for meaningful comparison across participating cohorts. The metagenomic profiles in this dataset originate from multiple cohorts and contain healthy individuals as well as patients with colorectal cancer.

Our aim is to reuse this data to run fedflow with two different FeatureCloud apps: singular value decomposition (SVD) and a random forest classifier with both centralized and federated data. For the federated case, we selected 5 cohorts of this colorectal cancer clinical and gut microbiome harmonized dataset and assigned one accession to each of 5 remote clients (accessions in data availability statement). The data of each cohort contain between 128 and 199 samples with batch-effect corrected metagenomic species abundances (for data processing please see Barbet *et al.* 2025) as well as age, gender, body mass index, and colorectal cancer status (total of 2547 features). In contrast, the centralized setting holds pooled data from all 5 cohorts (837 samples) and is executed via FeatureCloud on a single cloud instance.

The workflow is executed without manual intervention and illustrates how federated analyses can be embedded into standard workflow management systems using fedflow. Importantly, this analysis is not intended to assess biological performance or evaluate model accuracy, but simply to show incorporation of FeatureCloud in multi-step workflows. Accordingly, the random forest classifier was used as an illustrative federated application and was not subjected to hyperparameter optimization.

**Figure 2:**
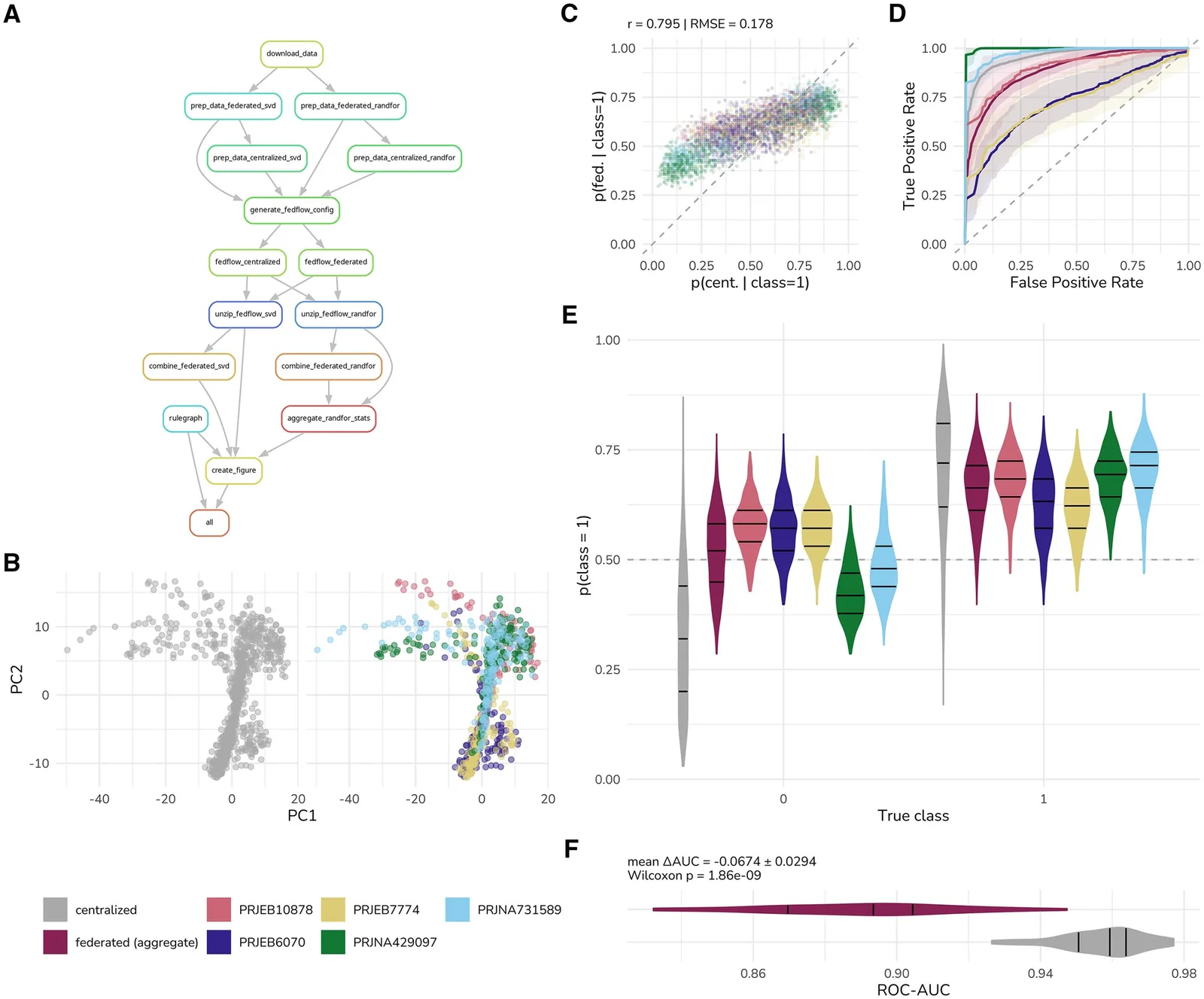
Example federated workflow and comparison of centralized and federated executions. (A) Rule graph of the Snakemake workflow including data preparation, centralized and federated execution branches, post-processing, and figure generation. The proposed orchestration framework enables the incorporation of federated learning with FeatureCloud apps in such workflows. (B) Two-dimensional projections from singular value decomposition under centralized and federated execution, demonstrating agreement between both approaches. (C) Comparing the predicted class probabilities for disease state from centralized and federated random forest classifiers shows strong correlation between execution settings. (D) Performance is evaluated with ROC curves for centralized and federated classifiers and indicates comparable results. (E) By separating predicted disease state probabilities by the underlying true class we observe consistent predictive trends, although with reduced class separation in the federated setting.

The pipeline downloads the data and prepares the input for each tool, e.g. partitioning samples for classification into training and test sets using repeated 80/20 resampling for held-out model evaluation. Next, fedflow runs both FeatureCloud apps with federated and centralized execution branches, respectively, and finally results are gathered and visualized (Fig. 2).

First, we compared centralized and federated SVD. For this analysis all federated data sets need to provide the same feature space and encoding of categorical variables. Metagenomic abundances and continuous clinical variables were centered and scaled to unit variance, and gender was included in binary encoding. Variable standardisation was performed by the SVD implementation in FeatureCloud (Hartebrodt 2022, Hartebrodt *et al.* 2022). Input values might therefore differ depending on whether standardisation was performed on the entire dataset or subsets thereof. For federated execution, this implementation uses multiple iterations to exchange covariance matrices and eigenvalues between participants and results in projections of the local data onto final vectors (Hartebrodt *et al.* 2022). Given matched preprocssing and sufficient convergence of iterations, federated SVD is expected to approximate the centralized decomposition of the same feature matrix. Indeed, the low-dimensional embeddings obtained from federated execution closely match those from centralized analysis, indicating that federated computation reproduces the global structure of the data without data centralization (Fig. 2B). Differences between projections are minimal ($\left||x_{\mathrm{cent}}-x_{\mathrm{fed}}||=(1.20\pm 0.85)\times 10^{-3}(\mu \pm \sigma \right)$) and attributable to execution context or numerical precision rather than workflow logic.

Next, we evaluated a random forest classifier under centralized and federated configurations. The FeatureCloud app implementing federated random forests uses scikit-learn’s RandomForestClassifier (Pedregosa *et al.* 2011, Späth and Matschinske 2023). In this setting individual models are trained locally and their decision trees and split rules are aggregated by the coordinator node into a global ensemble model, which is redistributed to all participants. Predictions on the test data are then performed using the global forest.

In our example, predicted class probabilities from both approaches are strongly correlated (Fig. 2C). Classification performance, assessed via ROC curves, indicates that the federated model recovers the performance of a model trained on pooled data (Fig. 2D), although differences in the performance between cohorts exist. These differences are expected, as cohort-specific composition, residual batch effects, class imbalance, or covariate shifts can influence the locally learned trees and therefore the aggregated federated ensemble. ROC curves represent the average performance across 30 repeats of the 80/20 train-test split with shaded areas indicating the standard deviation of the mean.

Further, when separating predicted probabilities by true class (health status) federated model training show comparable predictive trends, albeit with reduced separation between classes compared to pooled data (Fig. 2E). This suggests that additional model calibration may be necessary in federated settings to achieve well-separated class probability distributions.

A paired Wilcoxon signed-rank test comparing the difference in ROC-AUC between centralized and federated models across 30 repeats shows a significant difference, indicating that the federated classifier does not reproduce the full performance of the centralized one without further calibration (Fig. 2F; Δroc-auc −0.0674 ± 0.0294(μ ± σ), P<0.001).

The models were trained with 100 estimators and 5-fold cross validation was used outside of federation to test for model generalisation (roc-auc 0.956 ± 0.014(μ ± σ)). We did not perform hyperparameter tuning because this classifier served as a workflow demonstration rather than a performance-optimized analysis of federated versus centralized approaches. In a practical federated study, hyperparameter selection could be carried out by orchestrating repeated federated runs with fedflow using different parameter settings and selecting models based on aggregated validation metrics, without transferring raw data between sites.

In a separate analysis we tested how fedflow behaves with varying number of clients and input data and found that the orchestration scales approximately linearly with participant count (see Supplementary Material; Fig. S1, available at supplementary material  *Bioinformatics Advances* online).

### 3.1 Conclusion and limitations

In this work we introduce fedflow, an orchestration layer to automate FL tasks on the FeatureCloud platform. Previously, such tasks required manual intervention of all participants on a graphical interface. With fedflow, FeatureCloud runs can be distributed across cloud instances or other VMs, and integrated in automated reproducible workflows.

However, fedflow has limitations that we can group into three categories. Firstly, technical constraints which concern e.g. automatic provisioning of remotes machines. This is currently implemented for instances based on the debian operating system or any derivatives using the apt package manager. To install dependencies on other systems a custom provisioning script needs to be supplied alongside the configuration. Further, the underlying API does not include the creation of users on the FeatureCloud platform and registration of their physical sites. Rather, these steps need to be performed once via the platform’s website. Another omission from our API concerns the possibility to chain FeatureCloud apps during project definition on the web interface. With fedflow the execution of multiple FL tasks can instead be handled by established workflow management systems.

Secondly, fedflow offers limited security and relies on trust assumptions. For client orchestration, fedflow utilizes remote execution via ssh and authenticated interaction with the FeatureCloud platform. As a consequence, deployments require a trusted execution environment and careful credential management. Potential risks include unauthorized access through compromised ssh keys or platform credentials, misconfigured remote permissions, and post-approval modification of workflow code or runtime parameters. While raw input data are not transferred between participants by the federated apps, this alone does not eliminate privacy risks.

Additional security measures could include client-side disabling of interactive access, restriction of remote execution to specific scripts or binaries, and using short-lived credentials. In this regard, fedflow is not intended to be a secure multi-party computation framework, but rather a practical way to use the FeatureCloud platform. Automation frameworks such as the one presented here are indispensable, especially for iterative analyses or simulations of FL.

More generally, federated learning may still expose information through exchanged intermediate results or final model objects, depending on the underlying app. For example, covariance-based summaries, model updates, or aggregated tree ensembles could in principle leak information about local data distributions, especially for small cohorts or in the presence of other metadata. More advanced privacy-preserving techniques such as secure aggregation, differential privacy, homomorphic encryption, or secure multi-party computation (reviewed in Wan *et al.* 2022) are not implemented by fedflow itself, but can be incorporated in the executed FeatureCloud apps.

Lastly, there are operational and scalability considerations. To characterize the overhead of the orchestrator itself, we conducted a benchmark study with a lightweight federated application across up to 64 clients and varying input data sizes (Supplementary Material, available at supplementary material  *Bioinformatics Advances* online). These measurements indicate approximately linear growth of runtime and memory usage with client count but without becoming prohibitive.

To increase robustness we implemented automatic retries upon transient connection errors. However, fedflow does not include any checkpointing or fault tolerance, e.g. in the case of individual VMs failing during execution. In its current state, fedflow depends on undocumented API endpoints of FeatureCloud. Hence, future evolution of the platform or the local docker controller might require significant updates to how clients communicate with the central components.

## Supplementary Material

vbag255_Supplementary_Data

## Data Availability

The software described in this article and its documentation, as well as the example workflow are available at https://github.com/W-L/fedflow. Fedflow, alongside its dependencies, can be installed with pip install fedflow-featurecloud. Data underlying the example workflow are available at the Recherche Data Gouv repository https://doi.org/10.57745/7IVO3E. A subset of this curated repository was used which includes the following BioProject accessions: PRJEB10878, PRJEB6070, PRJEB7774, PRJNA429097, PRJNA731589.
