# Supplementary material for: Fedflow: cloud orchestration for federated learning with the FeatureCloud platform
Source: Bioinform Adv. 2026 Sep 4;6(1):vbag255. doi: 10.1093/bioadv/vbag255 (PMC13561539; doi:10.1093/bioadv/vbag255)
Supplement: Supplementary file 1 [file vbag255_supplementary_data.pdf]

Supplementary Material, 2026
Application Note | **Software**

Supplementary Material

Fedflow: cloud orchestration for federated learning with the FeatureCloud platform

Lukas Weilguny[1,2,*], Niklas Probul[3], Yani Ren[1], Nicolas Pons[1], Jan Baumbach[3, 4] and Mathieu Almeida[1]

[1]Université Paris-Saclay, INRAE, MGP, F-78350, Jouy-en-Josas, France
[2]Université Paris-Saclay, INRAE, Micalis Institute, F-78350, Jouy-en-Josas, France
[3]Institute for Computational Systems Biology, Universität Hamburg, Hamburg, Germany
[4]Department of Mathematics and Computer Science, University of Southern Denmark, Odense, Denmark

Scalability of fedflow orchestration

To assess the operational scaling of the fedflow orchestration independently of the federated algorithm, we implemented a dedicated benchmark workflow using the FeatureCloud `mean-app`, which computes a federated average of local numerical input values. This choice keeps the time spent executing the federated app small relative to the orchestration steps performed by fedflow and therefore allows us to characterize its scaling behaviour. Further, we provisioned all virtual machines (1 CPU core and 1 GB of RAM each, Ubuntu 24.04) in a preliminary step before running the benchmark to avoid confounding the measurements with installation of software. The orchestration was executed on a machine with 8 cores and 16 GB of RAM, using fedflow version 0.0.5. The benchmark varies both the number of participating clients and the amount of input data per client. Specifically, we executed fedflow with 1, 2, 4, 8, 16, 32, and 64 clients and with per-client input sizes of 10, 100, 1000, and 10000 random floating-point values, using 5 repeated runs for each condition.

Across conditions, total wall-clock time, CPU time, and memory usage increase approximately linearly with the number of clients (Fig. S1A–C). In contrast, at a fixed client count, varying the amount of input data per participant only shows a minor effect (Fig. S1D). This is consistent with our goal of using the lightweight federated mean to ensure that the measured runtime is dominated by orchestration overhead rather than by the underlying federated computation.

Detailed phase-level measurements show that the largest contributions to runtime come from clients joining a FeatureCloud project and contributing their local data (Fig. S1E). Whereas these steps are executed in a threaded way (in our benchmark on a machine with 8 cores) they are responsible for a large part of API interactions. Accordingly, these steps must be offset in time to limit the load placed on the central FeatureCloud server. By contrast, monitoring of the project execution, i.e. the actual computation of the federated algorithm, remains comparatively stable across client counts. An example run with 64 clients illustrates how the total runtime is distributed across the individual phases (Fig. S1F).

Overall, these measurements suggest that fedflow scales in an approximately linear way with the number of participants while maintaining moderate memory usage. Because the benchmark intentionally uses a minimal federated app, these results should be interpreted as orchestration overhead and not as a general proxy for end-to-end runtime. Using more computationally demanding FeatureCloud applications will necessarily increase the total runtime of each execution, but the relative contribution of the orchestration overhead is expected to remain similar.

The code for this scalability workflow is available alongside fedflow at `https://github.com/W-L/fedflow/tree/main/analysis/workflow_benchmarks` and can be executed with snakemake. To produce the results shown here we utilized the HPC facilities of the National Network of Computing Resources of the Institut Français de Bioinformatique (IFB), funded by the Programme d'Investissements d'Avenir (PIA), grant Agence Nationale de la Recherche number ANR-11-INBS-0013.

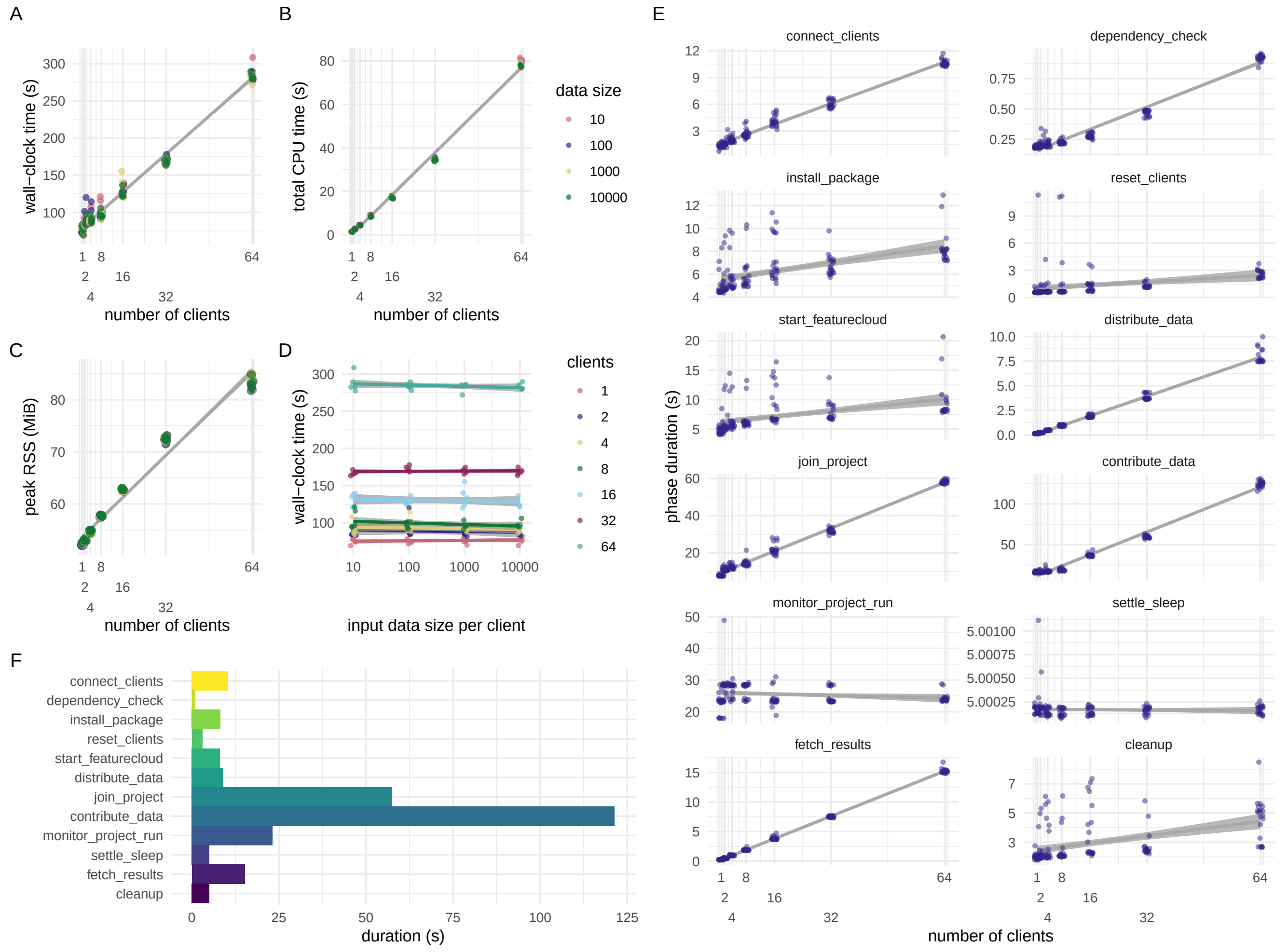

Supplementary Figure S1 Scaling benchmark of fedflow using the FeatureCloud `mean-app`. In all panels, points correspond to individual repeated runs and lines indicate linear trends with shaded confidence bands. A) The total wall-clock time as a function of number of clients shows an approximately linear increase. Data points are colored by the input data amount per client. B) Only a portion of total runtime is spent on CPU execution, but this proportion equally increases with client count. The legend here also applies to panels A and C. C) The peak memory usage (measured as resident set size, RSS) for runs with increasing number of participants remains low even at 64 clients. D) In this benchmark the amount of input data does not significantly affect the total runtime. E) Separating the runtime into individual orchestration phases shows that their duration increases with the number of clients. The most time consuming steps involve the largest proportion of API calls to the FeatureCloud platform. F) An example runtime breakdown for one run with 64 clients illustrates how total runtime is dominated by clients joining a FeatureCloud project and contributing their local data.
